# Liver X receptor agonist treatment significantly affects phenotype and transcriptome of APOE3 and APOE4 *Abca1* haplo-deficient mice

**DOI:** 10.1371/journal.pone.0172161

**Published:** 2017-02-27

**Authors:** Alexis Y. Carter, Florent Letronne, Nicholas F. Fitz, Anais Mounier, Cody M. Wolfe, Kyong Nyon Nam, Valerie L. Reeves, Hafsa Kamboh, Iliya Lefterov, Radosveta Koldamova

**Affiliations:** Department of Environmental and Occupational Health, University of Pittsburgh, Pittsburgh, PA, USA; Nathan S Kline Institute, UNITED STATES

## Abstract

ATP-binding cassette transporter A1 (ABCA1) controls cholesterol and phospholipid efflux to lipid-poor apolipoprotein E (APOE) and is transcriptionally controlled by Liver X receptors (LXRs) and Retinoic X Receptors (RXRs). In APP transgenic mice, lack of *Abca1* increased Aβ deposition and cognitive deficits. *Abca1* haplo-deficiency in mice expressing human APOE isoforms, increased level of Aβ oligomers and worsened memory deficits, preferentially in APOE4 mice. In contrast upregulation of *Abca1* by LXR/RXR agonists significantly ameliorated pathological phenotype of those mice. The goal of this study was to examine the effect of LXR agonist T0901317 (T0) on the phenotype and brain transcriptome of APP/E3 and APP/E4 *Abca1* haplo-deficient (APP/E3/Abca1^+/-^ and APP/E4/Abca1^+/-^) mice. Our data demonstrate that activated LXRs/RXR ameliorated APOE4-driven pathological phenotype and significantly affected brain transcriptome. We show that in mice expressing either APOE isoform, T0 treatment increased mRNA level of genes known to affect brain APOE lipidation such as *Abca1* and *Abcg1*. In both APP/E3/Abca1^+/-^ and APP/E4/Abca1^+/-^ mice, the application of LXR agonist significantly increased ABCA1 protein level accompanied by an increased APOE lipidation, and was associated with restoration of APOE4 cognitive deficits, reduced levels of Aβ oligomers, but unchanged amyloid load. Finally, using Gene set enrichment analysis we show a significant APOE isoform specific response to LXR agonist treatment: Gene Ontology categories “Microtubule Based Process” and “Synapse Organization” were differentially affected in T0-treated APP/E4/Abca1^+/-^ mice. Altogether, the results are suggesting that treatment of APP/E4/Abca1^+/-^ mice with LXR agonist T0 ameliorates APOE4-induced AD-like pathology and therefore targeting the LXR-ABCA1-APOE regulatory axis could be effective as a potential therapeutic approach in AD patients, carriers of *APOEε4*.

## Introduction

The inheritance of *APOEε4* allele is the major genetic risk factor for late-onset Alzheimer disease (LOAD) [1]. AD patients, carriers of *APOEε4* allele show earlier onset of the disease and higher amyloid load. AD mouse models expressing human APOE isoforms to a large extent recapitulate amyloid phenotype and cognitive deficits [2–4]. The mechanism by which APOE4 affects the pathogenesis of AD remains poorly understood and it is still unclear if *APOEε4* allele confers insufficient protection against beta-amyloid (Aβ) or if it has deleterious effects [5, 6]. Compared to *APOEε3* carriers, the higher incidence of LOAD and an increased Aβ deposition in *APOEε4* carriers might be a result of lower brain APOE protein levels, or its lower lipidation [6]. In addition to *APOEε4*, other low prevalence functional genetic variants are proposed as risk modifiers [7].

ATP-binding cassette transporter A1 (ABCA1) controls cholesterol and phospholipids efflux to lipid-poor apolipoproteins and is essential for the formation of HDL in periphery and HDL-like particles in brain [8]. While rare *ABCA1* variants have been shown to affect plasma HDL and AD risk [9], genome wide association studies (GWAS) have produced conflicting reports on the associations of common *ABCA1* genetic variants with LOAD (reviewed in [8]). A recent study reported that a rare variant of ABCA1 (N1800H) resulting in a loss-of-function is associated with high risk of AD and cerebrovascular disease in a large cohort from the Danish general population [10]. This variant, found in 1:500 individuals, has a well-established effect on plasma HDL and cholesterol efflux, and is associated with low plasma levels of APOE [10].

In transgenic mice expressing human APP, global deletion of *Abca1* translates into substantially decreased amounts of APOE and increased amyloid deposition [11–13]. In contrast, *Abca1* overexpression decreases amyloid pathology [14]. The effect of *Abca1* gene dose on AD-like phenotype was examined in old APP transgenic mice expressing mouse *Apoe* and the results demonstrated that presence of one copy of *Abca1* in the mouse genome significantly worsened memory deficits in correlation with an increased amount of Aβ oligomers [15]. Interestingly, in middle-aged APP mice expressing human APOE isoforms, *Abca1* haplo-deficiency differentially affected the phenotype of APP/E4 mice in that it decreased Aβ clearance, plasma HDL level and increased amyloid load preferentially in APP/APOE4 mice [3]. This suggests that APOE4 confers less resistance to additional genetic defects and increasing APOE4 level may be beneficial in ameliorating AD phenotype.

The expression of *ABCA1* in humans and rodents is transcriptionally controlled by Nuclear receptors Liver X Receptors (LXRα and LXRβ). LXRα/β are transcription factors that form obligate heterodimers with Retinoid X Receptors (RXRs). Previous studies demonstrated that treatment with synthetic LXR ligands decreased Aβ burden, increased its clearance in APP expressing mice and decreased inflammatory reactions in brain [16–18]. Since the diverse effects of activated LXRs/RXRs heterodimers in brain and periphery have been ascribed to changes in transcriptome [19, 20] and their activation status can be easily monitored and manipulated, the application of synthetic small molecule LXR/RXR ligands has also been suggested as a rational therapeutic approach in the treatment of AD [17, 19–23]. We have shown that synthetic RXR agonist, bexarotene, restored memory deficits in APP/PS1 transgenic mice expressing either *APOE3* or *APOE4* and in correlation with the improved memory performance significantly decreased the amount of soluble oligomers in the brain [24]. Boehm-Cagan et al. confirmed the effect on cognition in non-transgenic WT type mice expressing human APOE isoforms [25]. However, whereas our data showed that bexarotene treatment significantly decreased Aβ level in the interstitial fluid of APP/PS1 transgenic mice expressing either APOE isoform, there was no effect on amyloid plaque load [26]. Thus our findings were in contrast to the original report by Cramer et al. [24] showing rapid plaque clearance, and in agreement with other studies [27–30]. Our recent data suggest that the effects of activated LXRs/RXRs extend beyond cholesterol and phospholipids efflux, improving neuronal differentiation and adult neurogenesis in *APOE4* and *APOE3* mice [31, 32].

The aim of this study was to examine changes in brain transcriptome of *Abca1* heterozygous APP/E3 and APP/E4 mice treated with LXR ligand T0901317 and correlate the results to the phenotype.

## Materials and methods

### Chemicals and reagents

T0901317 (T0) was purchased from Cayman Chemical (Ann Arbor, MI). All other materials were purchased through Fisher Scientific, unless otherwise noted.

### Animals and diet

All animal experiments were approved by the University of Pittsburgh Institutional Animal Care and Use Committee and carried out in accordance with PHS policies for use of animals in research. APP/PS1ΔE9 mice [33] and Abca1^+/-^ heterozygous mice were purchased from The Jackson Laboratory (Bar Harbor, ME). Human *APOE4*^+/+^ and *APOE3*^+/+^ targeted replacement mice (*APOE*-TR) were originally purchased from Taconic (Germantown, NY). All mice that were either purchased or bred for at least ten generations were on C57BL/6 genetic background. APP/PS1ΔE9/APOE4^+/+^/Abca1^+/-^ and APP/PS1ΔE9/APOE3^+/+^/Abca1^+/-^ (referred to as APP/E4/Abca1^+/-^ and APP/E3/Abca1^+/-^ respectively) as well as non–transgenic, expressing endogenous APP, littermates (referred to as E4/Abca1^+/-^ and E3/Abca1^+/-^) were bred as previously described [3]. At five months of age, 104 APP transgenic and non–transgenic controls (APP/E4/Abca1^+/-^, 11 females and 15 males; APP/E3/Abca1^+/-^, 12 females and 17 males; E4/Abca1^+/-^, 14 females and 9 males; E3/Abca1^+/-^, 13 females and 13 males) were randomly assigned to vehicle (control) or T0 fed diet.

Each diet was prepared as previously described [34]. Briefly, T0 was dissolved in dimethyl sulfoxide (DMSO), Cremophor (Sigma–Aldrich, St. Louis, MO), then double distilled water (final 0.03% DMSO in prepared food), mixed with milled standard chow (Prolab® Isopro® RMH 3000, 5P76, LabDiet®, St. Louis, MO) and divided into daily portions. The diet was dried in order to achieve a 0.028% (w/w) T0 drug concentration and a dosage of 20 to 25 mg T0/kg mouse/day. Standard chow for the vehicle group was prepared as described, but only containing DMSO and Cremophor. Mice were kept on the corresponding diets for 28 days and assessments (behavior, immunohistochemistry, gene expression and Western blotting) performed at 6 months of age. Age and gender matched non–transgenic littermates were used as controls for behavior experiments.

### Behavioral testing

#### Novel object recognition

Novel object recognition (NOR) was performed as previously described with modifications [35]. On day one, mice were acclimated to the behavioral arena (White plastic box, 40 cm x 40 cm x 30 cm) for ten minutes. Twenty-four hours following acclimation, mice were placed in the center of the arena with two similar objects (Tower of Lego® bricks 8cm x 3.2cm, built in white, blue, yellow, red and green bricks) and allowed to explore the objects for two trials lasting five minutes each, with a five minute inter-trial interval. The similar objects were located in the east and west quadrant and spaced equidistant from the arena walls. Twenty-four hours following the habituation, one object was replaced with a novel object (large metal bolt and nut of similar size). Mice were placed in the arena and allowed to explore the objects for ten minutes. Exploratory visit was defined as the mouse sniffing, climbing on, or touching an object or within three centimeters while facing an object. Exploration time was recorded and scored with ANY-maze software (Stoelting Co., Wood Dale, IL). The arena was cleaned with 70% ethanol between animals to eliminate olfactory cues. Exploration time was calculated by dividing time exploring the novel object by total time exploring objects. Animals exhibiting memory impairments spent less time exploring the novel object.

#### Contextual fear conditioning

Contextual fear conditioning (Equipment obtained from Stoelting Co., Wood Dale, IL) was performed as previously described with minor modifications [36]. Briefly, mice were placed in a conditioning chamber for two minutes, followed by 30 seconds of tone representing the conditioned stimulus (Sound, 2800 Hz; Intensity, 85 dB). At the end of the tone, mice received a foot shock (0.7 mA) for two seconds through the floor of the chamber. The cycle was repeated once more. At the end of the second cycle, the mice remained in the chamber for 30 seconds before returning to their housing cages. Twenty-four hours after the training phase, contextual fear conditioning was assessed and consisted of measuring freezing behavior for five minutes in the original conditioning chamber. Twenty-four hours after the contextual phase, freezing behavior during the cued fear conditioning was assessed and consisted of placing mice in a novel context for two minutes (plain gray walls replace by black and white stripped walls), followed by exposure to the conditioned stimulus for three minutes. Freezing behavior was defined as the absence of movement except for respiration. Freezing behavior was recorded using ANY-maze software and calculated as percent freezing of the total time spent in the chamber.

### Animal tissue processing

Mice were anesthetized with Avertin (Intraperitoneal injection; 250 mg/kg of body weight). Blood was collected through cardiac puncture followed by transcardial perfusion with 25 ml of cold 0.1 M phosphate buffered saline (PBS), pH 7.4 [36]. Brains were removed and divided into hemispheres. One hemisphere was dissected into cerebellum, subcortical region, hippocampus and cortex and snap frozen on dry ice. The second hemisphere was fixed in 4% paraformaldehyde at 4°C for 48 hours, and then stored in 30% sucrose.

### Histology and Immunohistochemistry

Histology, X-34 and 6E10 immunohistochemistry was performed as previously described [31, 36]. Brain hemispheres were removed from the 30% sucrose solution and embedded in HistoPrep™. Brain hemispheres were cut into 30 μm coronal sections. Six sections starting at the formation of the dentate gyrus (-1.2mm from Bregma) and separated by 450 μm were used for immunohistochemistry. Sections were stored in glycol-based cryoprotectant at -20°C until staining.

For X-34 staining, sections mounted on positively charged glass slides were washed in PBS and treated with 1,4-bis(3-carboxy-4-hydroxyphenylethenyl)-benzene (X-34) for 10 min each. Slides were destained with 0.2% NaOH in 80% ethanol for 3 min and washed with PBS before and after destaining.

For 6E10 staining, sections adjacent to those used for X-34 were immunostained with biotinylated 6E10 antibody (803009, Biolegend, San Diego, CA). Antigen retrieval was done on free-floating sections with 70% formic acid. Blocking of endogenous peroxidases and avidin-biotin followed antigen retrieval. Then, sections were incubated with 6E10 biotin-labeled antibody (1:1000) overnight at 4°C and developed with Vectastain ABC Elite kit and DAB substrate (Vector Laboratories, Burlingame, CA). After staining, sections were coverslipped with Permafluor.

All sections were examined under the microscope using the Nikon Eclipse 90i at 10× magnification. For quantitative analysis, percent positive staining was defined as the percent area covered by X-34 or 6E10 staining using NIS Elements software (Nikon Instruments Inc., Melville, NY).

### Western blotting

To prepare lysate for both Western blotting and ELISA, frozen cortices were homogenized in a glass Dounce containing tissue homogenization buffer (250 mM sucrose, 20 mM Tris base, 1 mM EDTA, and 1 mM EGTA (Sigma–Aldrich, St. Louis, MO, 1 ml per 100 mg of tissue) and protease and phosphatase inhibitor cocktail (Roche, Indianapolis, IN). The Bradford assay was used to determine protein concentration of all samples. The supernatant of the initial homogenate (TBS extract) was used to determine soluble APOE (EMD Millipore, Temecula, CA) and APOJ (Santa Cruz Biotechnology, Dallas, TX) concentration. The pellet was re-suspended, sonicated and spun with RIPA buffer containing protease and phosphatase inhibitors. 30 μg of total protein was mixed with Tris-Glycine denaturing loading buffer, loaded, and electrophoresed on 10% Tris-Glycine or 4–12% Bis-Tris gels. On nitrocellulose membranes ABCA1 was detected using polyclonal antibody, ab7360 (Abcam, Cambridge, MA), and APPfl with 6E10 antibody. β-Actin served as a loading control for all Western blots and detected using a monoclonal antibody (Sigma-Aldrich, St. Louis, MO). Membranes were incubated with respective secondary antibodies conjugated to horseradish peroxidase, and visualized by enhanced chemiluminescence, Plus-ECL (PerkinElmer, Waltham, MA). Blots were imaged using the chemiluminescent setting on the Amersham Imager 600 (GE Healthcare Life Sciences, Marlborough, MA). All bands were quantified by densitometry (ImageQuant, version 5.2; GE Healthcare) and normalized to β-Actin. To quantify APPfl and ABCA1, bands were normalized to respective vehicle groups. Quantification of APOE and APOJ is represented as fold of vehicle treated APP/E3/Abca1^+/-^ mice.

### Native PAGE

Native PAGE was performed according to a previously published protocol [37] with slight modifications. TBS brain extract was mixed with 2× non-denaturing loading buffer and resolved on Novex™ 4–20% Tris-Glycine gels. TBS brain extract from *Apoe*^ko^ mice was used as a negative control. Amersham™ HMW calibration kit was used as a native ladder (GE Healthcare, Marlborough, MA). Polyclonal anti-APOE (EMD Millipore, Temecula, CA) along with respective secondary antibody was used for incubation and developed as described for Western blot. Quantification of lipidated APOE is represented as the fold of respective vehicle groups.

### Aβ oligomer ELISA

Aβ oligomer ELISA was performed as previously published [36] with few modifications. We used a standard curve of Aβ_1–40_Ser26Cys dimer. 6E10 antibody was used as the capture antibody (10 μg/ml) to coat a 96 well Nunc MaxiSorp plate overnight at 4°C. After removing the antibody, the plate was washed with PBS and blocked with Block Ace for four hours. Following the removal of Block Ace, Aβ_1–40_Ser26Cys dimer standards and RIPA fraction from the cortex were diluted in EC buffer and loaded on the plate in duplicates. Biotinylated 6E10 antibody was used as the detection antibody (0.167 μg/ml) and incubated 4 hours at room temperature. The assay was developed with HRP-labeled streptavidin (1:30,000) for 1.5 hours at RT, followed by the use of the TMB Microwell Peroxidase Substrate System (KPL, Gaithersburg, MD). Plate was read on the SpectraMax i3 (Molecular Devices, Sunnydale, CA) at 650 nm. Final values were compared to Aβ_1–40_Ser26Cys dimer standard curve using linear regression analysis, normalized to the total protein concentration in each sample and expressed as ng Aβ/mg protein.

### RNA isolation, qPCR, and sequencing

RNA was isolated from the cortex and purified according to the RNAeasy mini kit manufacturer protocol (Qiagen, Valencia, CA) as previously described [32]. RNA quality was determined using the 2100 Bioanalyzer instrument (Agilent Technologies, Santa Clara, CA). Samples with a RIN > 8 were used for library generation and sequencing (mRNA Library Prep Reagent Set; Illumina, San Diego, CA) on the Illumina HiSeq2000 instrument at the Functional Genomics Core, University of Pennsylvania, Philadelphia, PA (http://fgc.genomics.upenn.edu/). Subread (v1.5.0, http://subread.sourceforge.net) was used to align sequencing reads to the mouse genome (mm9). EdgeR package (v3.14.0) in R environment (v3.2.4) was used to analyze the differential gene expression. qPCR assays were performed using TaqMan™ Gene Expression Assay or Power SYBR® Green PCR Master Mix (Applied Biosystems, Foster City, CA). cDNA was synthesized using EcoDry™ Premix, Random Hexamers (Clontech, Mountain View, CA).

### Functional annotation clustering and analysis

Functional annotation clustering was performed using two different bioinformatics databases. We first used Database for Annotation, Visualization and Integrated Discovery (DAVID; http://david.abcc.ncifcrf.gov/; Huang et al., 2009) to determine gene ontology (GO) terms. Additional analysis with data from EdgeR output tables was performed using Gene set enrichment analysis (GSEA v2.2.2, https://www.broadinstitute.org/GSEA) with a gene matrix set for Biological Process (BP) (c5.bp.v5.1.symbols.gmt) [38, 39].

### Statistical analysis

All results are reported as means ± SEM. With the exception of RNA-seq results, all data were analyzed by two-way ANOVA for genotype and treatment factors followed by Sidak’s post hoc test or t-test. Unless otherwise indicated, all statistical analyses were performed in GraphPad Prism, version 6.0. Significance was determined as p < 0.05.

## Results

### Pharmacological activation of LXR/RXR transcription factors improves cognitive performance of *Abca1* haplo-deficient APP/E4 mice

**Fig 1:**
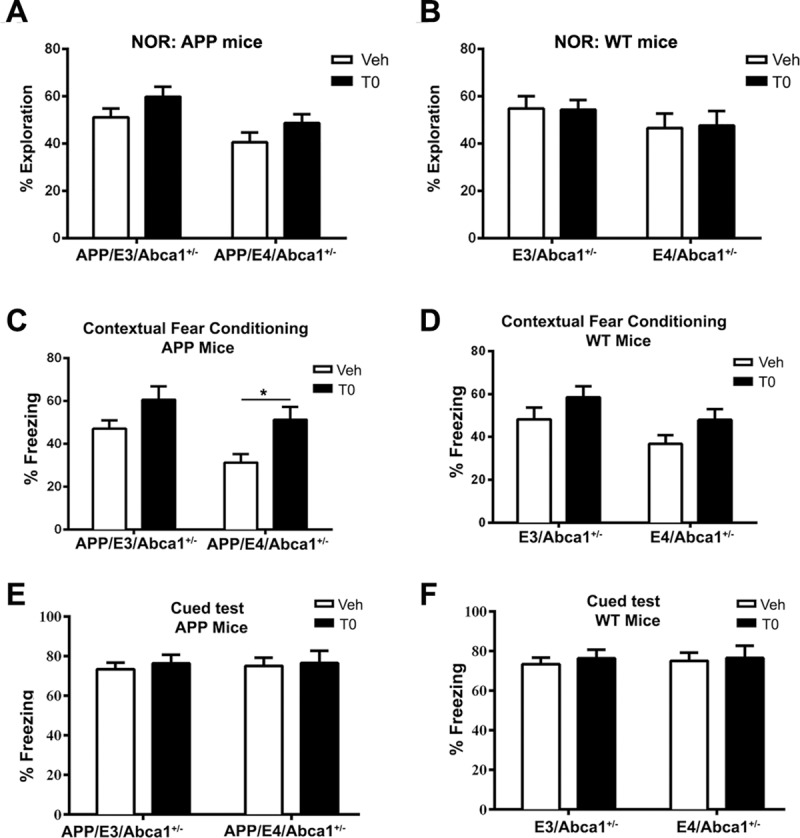
T0 treatment restores cognition in APP/E4/Abca1^+/-^ mice. 5-month-old APP/E3/Abca1^+/-^, APP/E4/Abca1^+/-^ and non-transgenic mice were treated with T0 and vehicle (Veh) for one month and assessed at 6 months of age. Cognitive function was evaluated with novel object recognition (A and B) and contextual fear conditioning behavioral paradigms (C and D). **A,** T0 affected the performance of APP transgenic mice in the novel object recognition test. Analysis by two-way ANOVA shows no interaction between *APOE* genotype and T0 treatment with a significant main effects of *APOE* genotype (F(1, 51) = 7.44, p < 0.01) and T0 treatment (F(1, 51) = 4.45, p< 0.05). **B,** T0 treatment did not affect the performance of non-APP littermates. Effects of *APOE* genotype (F(1, 45) = 1.9) and T0 treatment (F(1, 45) = 0.002). **C,** LXR agonist significantly improved the performance of APP/E4/Abca1^+/-^ mice in contextual fear conditioning paradigm. Analysis by two-way ANOVA shows no interaction between *APOE* genotype and T0 treatment and significant main effects of T0 treatment (F(1, 51) = 5.94, p = 0.018) and *APOE* genotype (F(1, 51) = 10.6, p = 0.002). Sidak’s multiple comparison test shows a significant difference between T0 and vehicle treated APP/E4/Abca1^+/-^ mice (*, p < 0.05). **D,** T0 also affected the behavior of non-APP controls in the contextual fear conditioning behavior paradigm. Analysis by two-way ANOVA shows no interaction and significant main effects of T0 treatment (F(1, 45) = 4.47, p = 0.03) and *APOE* genotype (F(1, 45) = 4.49, p = 0.04). T0 had no effect on APP (E) and non-APP mice (F) during the cued phase of fear conditioning. For all panels, N = 11–15 male and females mice per group. Data represented as means ±SEM.

We have previously demonstrated that *Abca1* deficiency differentially affects AD-like phenotype in mice expressing human *APOE4* or *APOE3*. To determine if ligand activated LXR can alleviate cognitive deficits in APP/E4/Abca1^+/-^ mice, we treated five month old APP/E4/Abca1^+/-^ mice with T0 for one month and the phenotype was examined at the age of six months. It should be noted that at the start of the treatment, the amyloid plaques are already present therefore the treatment should be considered therapeutic. First, we examined changes in cognitive function following T0 treatment in a novel object recognition paradigm. As seen in Fig 1A, analysis by two-way ANOVA revealed a significant main effect of *APOE* genotype [F(1, 51) = 7.44, p < 0.01] and T0 treatment [F(1, 51) = 4.45, p< 0.05]. In contrast, neither LXR ligand treatment nor APOE isoform had an effect on non-transgenic littermates (Fig 1B). To confirm the effect seen in novel object recognition, we used contextual fear conditioning paradigm that tests hippocampal-associated learning. As shown on Fig 1C, analysis by two-way ANOVA shows no interaction and significant main effects of T0 treatment (F(1, 51) = 5.94, p = 0.018) and *APOE* genotype (F(1, 51) = 10.6, p = 0.002). Sidak’s multiple comparison post-hoc test revealed a significant difference between T0 and vehicle treated APP/E4/Abca1^+/-^ mice (p < 0.05). Interestingly, in difference to novel object test, LXR ligand treatment and *APOE* genotype had a significant main effect on the performance of WT controls (Fig 1D). Cued test demonstrated no effect of T0 and *APOE* genotype confirming that the effect of T0 is reflected by hippocampal-associative memory (Fig 1E and 1F). Thus, the conclusion from these experiments is that LXR ligand treatment significantly improves cognition of APP/E4/Abca1^+/-^ mice.

### Ligand activated LXR/RXR do not affect amyloid plaque level but significantly decrease soluble Aβ oligomers in APP/E4/Abca1^+/-^ mice

**Fig 2:**
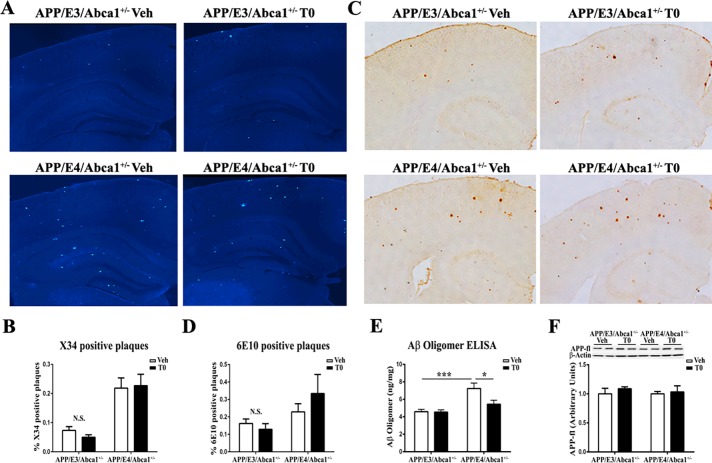
T0 treatment significantly decreased soluble Aβ oligomers, but not amyloid plaque pathology in APP/E4/Abca1^+/-^ mice. Amyloid plaque pathology of mice shown on Fig 1 was assessed by immunohistochemistry and ELISA. **A,** Brain sections were stained with X-34 to visualize compact fibrillary amyloid plaques in vehicle and T0 treated APP/E3/Abca1^+/-^ and APP/E4/Abca1^+/-^ mice. Representative images of X-34 staining were captured at 10× magnification. **B,** X-34 positive amyloid plaques were analyzed by two-way ANOVA. There is no interaction between *APOE* genotype and T0 treatment and a significant main effect of *APOE* genotype (F (1, 55) = 34.7, p < 0.0001), but not of T0 treatment. N = 14–16 mice per group. N.S., not significant. **C,** Brain sections were stained with anti-Aβ antibody, 6E10, to visualize diffuse and compact (total) amyloid plaques in vehicle and T0 treated APP/E3/Abca1^+/-^ and APP/E4/Abca1^+/-^ mice. Representative images of 6E10 staining are shown (10× magnification). **D,** 6E10 positive amyloid plaque load analyzed by two-way ANOVA. There is no interaction between *APOE* genotype and T0 treatment and a significant main effect of *APOE* genotype (F(1, 19) = 4.41, p = 0.049), but not of T0 treatment. N = 5–6 mice per group. N.S., not significant. **E,** T0 treatment significantly decreases Aβ oligomers in APP/E4/Abca1^+/-^ mice. RIPA fraction was evaluated for soluble Aβ by Aβ oligomer ELISA. Analysis by two-way ANOVA revealed an interaction between *APOE* genotype and T0 treatment (F(1, 32) = 4.82, p = 0.036). Sidak’s post-test demonstrated a significant difference between vehicle treated APP/E3/Abca1^+/-^ and APP/E4/Abca1^+/-^ mice (***, p<0.001) and T0 and vehicle treated APP/E4/Abca1^+/-^ mice (*, p<0.05). N = 6–10 mice per group. **F,** T0 has no effect on full-length APP. For all panels the data are means ±SEM.

To examine if LXR/RXR agonist treatment can alleviate amyloid plaque pathology in APP/E3/Abca1^+/-^ and APP/E4/Abca1^+/-^ mice, brain sections were stained with X-34 to visualize compact fibrillary amyloid plaques. Representative images of X-34 staining in the cortex and hippocampus are shown in Fig 2A. Analysis by two-way ANOVA confirmed a significant main effect of *APOE* genotype but there was no effect of T0 treatment. To visualize diffuse and compact (total) amyloid plaques, brain sections were stained with anti-Aβ antibody 6E10. Representative images of 6E10 staining in the cortex and hippocampus are shown in Fig 2C. Similarly, to the results of X-34 staining, the analysis showed a significant main effect of *APOE* genotype on the total amyloid burden, regardless of the T0 treatment (Fig 2D). Next, we determined the effect of T0 on the level of soluble Aβ oligomers in the cortices of APP/E3/Abca1^+/-^ and APP/E4/Abca1^+/-^ mice (Fig 2E). We found a statistically significant interaction between *APOE* genotype and T0 treatment and a difference in the amount of Aβ oligomers in T0 and vehicle treated APP/E4/Abca1^+/-^ mice (Sidak’s post-hoc test p < 0.05). These changes were not a consequence of T0 effect on full length APP processing as its protein level was unchanged (Fig 2F). The conclusion is that LXR ligand T0 does not affect amyloid plaques but significantly decreases soluble Aβ oligomers that confirms our previous data on the effect of activated LXR/RXR on amyloid pathology [26].

### Genome-wide effects of activated LXR/RXR on brain transcriptome in APP/E3/Abca1^+/-^ and APP/E4/Abca1^+/-^ mice

**Fig 3:**
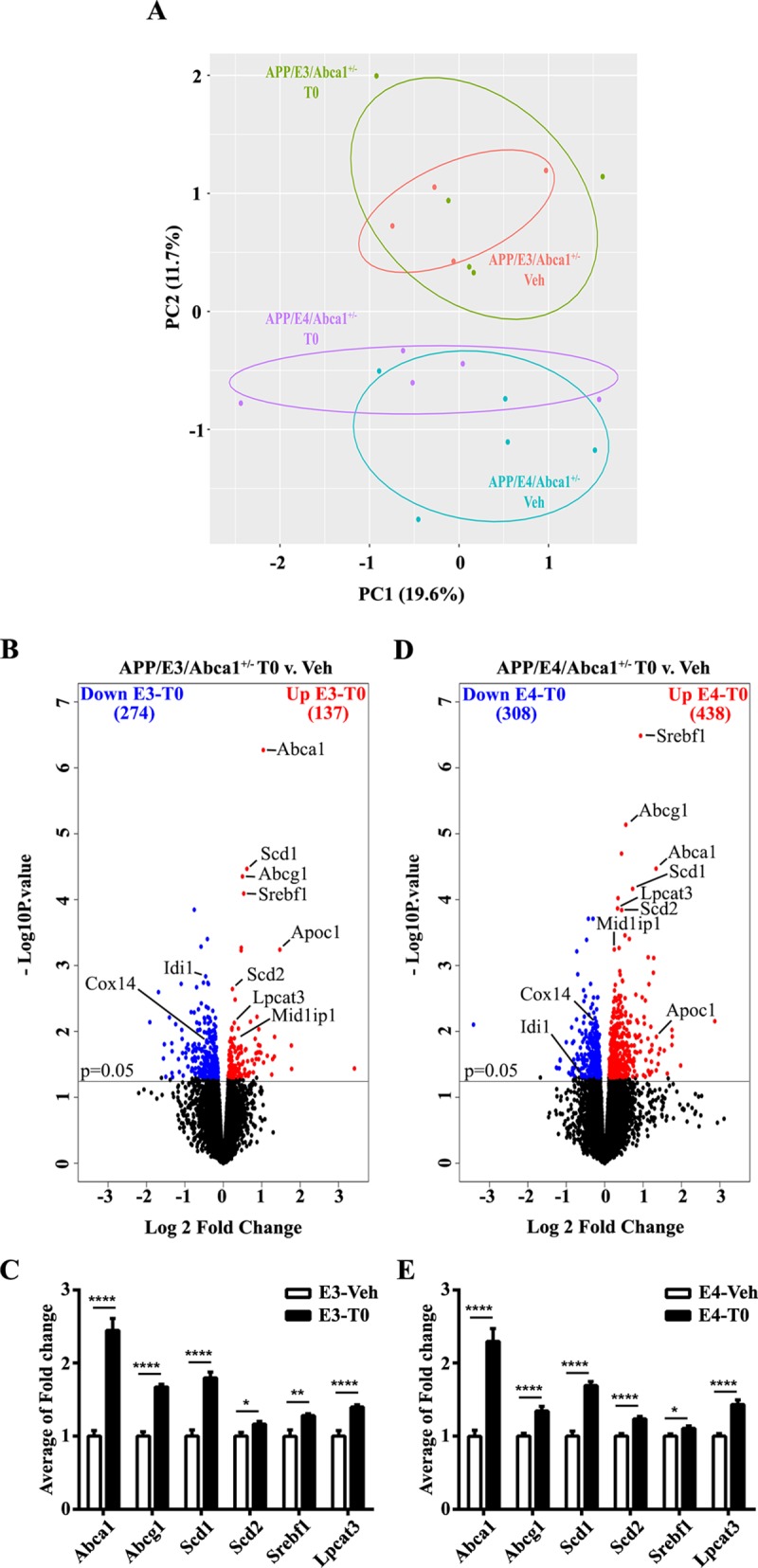
Transcriptional analysis of T0 treated six month old APP/E3/Abca1^+/-^ and APP/E4/Abca1^+/-^ mice. We used total RNA extracted from cortices of APP/E3/Abca1^+/-^ APP/E4/Abca1^+/-^ male mice treated with T0 or vehicle and shown on Fug.1 and 2. **A,** Principle component analysis (PCA) plot shows two dimensional comparison (PC1 vs PC2) of *APOE* genotype and T0 treatment in APP/E3/Abca1^+/-^ (4 mice for vehicle and 5 for T0 treatment) and APP/E4/Abca1^+/-^ mice (5 mice per group). **B** and **D,** The volcano plots show differential gene expression between T0 treated APP/E3/Abca1^+/-^ (**B**) and APP/E4/Abca1^+/-^ (**D**) mice when compared to their vehicle treated counterparts using EdgeR RNA-sequencing results analysis. Significant up-regulated genes are represented in red, significantly down-regulated genes are represented in blue; the cut off is at p<0.05. Up-regulated genes represent target genes of T0 treatment. **C** and **E,** qPCR validation of upregulated genes in T0 treated APP/E3/Abca1^+/-^ and APP/E4/Abca1^+/-^ mice from the volcano plot analysis. For C and E, N = 12 mice per group. qPCR values are mean ± SEM. Analysis were performed by student *t*-test. *, p<0.05, **, p<0.01, ****, p<0.0001.

To determine the effect of T0 treatment on the transcriptome of APP/E3/Abca1^+/-^ and APP/E4/Abca1^+/-^ mice shown on Figs 1 and 2, we performed RNA-seq. We used total RNA extracted from cortices of APP/E3/Abca1^+/-^ (4–5 mice per group) and APP/E4/Abca1^+/-^ (5 mice per group) male mice treated with T0 or vehicle and analyzed the sequencing datasets using edgeR v. 3.14.0 (http://bioconductor.org/). First, we evaluated the source of the variation in gene expression. We applied Principal Component Analysis (PCA) to process the abundance matrix of observed variables (static normalized expression level of genes across the genotypes and treatment) and to calculate Principal Components that account for most of the variance in the datasets. The scattered plot on Fig 3A is a two-dimensional (PC1 vs PC2) representation of T0 treatment and genotype. Interestingly, APP/E3/Abca1^+/-^ and APP/E4/Abca1^+/-^ mice formed two very distinct clusters encompassing the type of treatment. Thus it demonstrates that the effect of APOE isoform on gene expression is higher than the T0 treatment, yet APP/E4/Abca1^+/-^ mice were more responsive to pharmacological activation of LXR/RXR. Next we compared expression profiles of vehicle and T0 treated APP/E3/Abca1^+/-^ mice and identified a total of 411 differentially expressed genes: 137 up- and 274 down-regulated by T0 at a cut-off of p < 0.05 (Fig 3B). Using the same criteria, we found 746 differentially expressed genes in APP/E4/Abca1^+/-^ mice: 438 up- and 308 down-regulated following T0 treatment (Fig 3D). In mice expressing either APOE isoform, among common up-regulated genes known to affect brain lipoprotein metabolism and APOE lipidation were *Abca1*, *Abcg1* and *Lpcat3* (marked on the volcano plots shown on Fig 3B and 3D). Surprisingly, T0 treatment did not affect *APOE* mRNA level in mice expressing either isoform.

To examine biological categories affected by the treatment, we performed gene ontology analysis using DAVID. We submitted all significantly up-regulated genes (the number of genes is shown in red on Fig 3B and 3D) and downregulated genes (shown in blue on Fig 3B and 3D) at p < 0.05 cutoff. As visible from S1 Table, similarly upregulated by T0 in both APP/E3/Abca1^+/-^ and APP/E4/Abca1^+/-^ mice were processes related to lipid and cholesterol metabolism, DNA repair, and chromatin modifications. In contrast, there was little similarity between biological processes down regulated in *APOE3* and *E4* mice. As shown on S2 Table, in APP/E3/Abca1^+/-^ mice, significantly downregulated by T0 was GO term “innate immune response” including toll like receptor 3 (*Tlr3*), bone marrow stromal cell antigen 2 (*Bst2*), as well as interferon induced genes such as *Ifit1* and *Ifit3*, and *Oas2* and *Oasl2*. In contrast, uniquely and significantly downregulated in APP/E4/Abca1^+/-^ mice were transforming growth factor beta receptor signaling, cell differentiation, cell chemotaxis and synaptic transmission among others.

**Fig 4:**
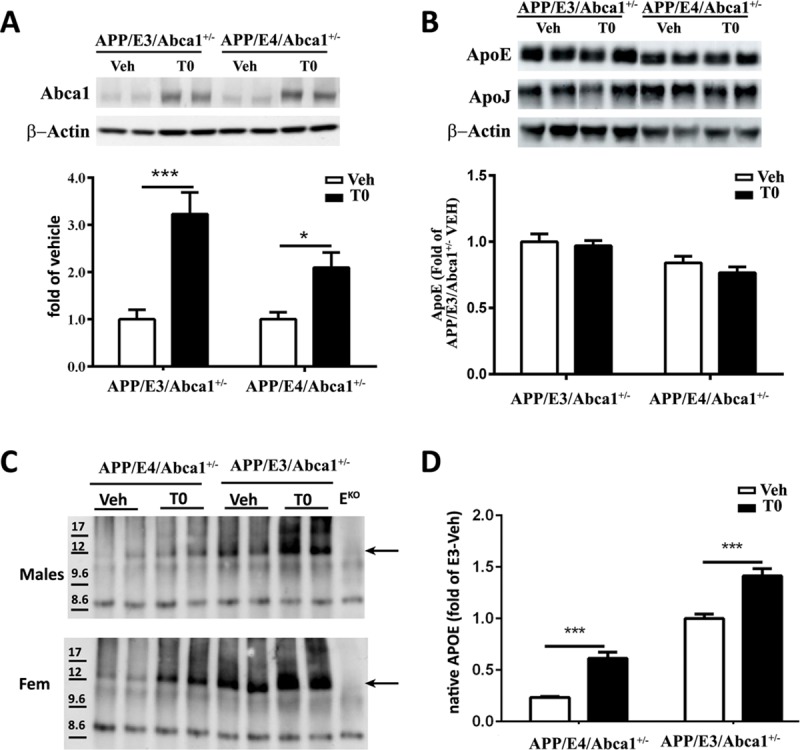
T0 treatment increases ABCA1 protein level and APOE lipidation. ABCA1, APOE and APOJ protein levels were determined by SDS-PAGE and APOE lipidation by Native PAGE. **A,** Representative image of ABCA1 protein level is shown above the graph. T0 significantly affected ABCA1 protein level. Analysis by two-way ANOVA shows no interaction between *APOE* genotype and T0 treatment. There is a significant main effect of T0 treatment (F(1, 34) = 26.12, p < 0.0001), but not of *APOE* genotype. Sidak’s post-test shows a significant difference between T0 and vehicle treated APP/E3/Abca1^+/-^ and APP/E4/Abca1^+/-^ mice. N = 8–10 mice per group. **B,** T0 treatment did not affect APOE or APOJ protein levels. N = 9–10 mice per group. **C,** APOE lipidation state in APP/E3/Abca1^+/-^ and APP/E4/Abca1^+/-^ mice. Representative images of APOE lipidation are shown: upper panel—male mice; lower panel—female mice (Fem). Arrows are indicative of lipidated APOE migrating at 12 nm. D, Quantification of native gel. Sidak’s post-test shows a significant difference between T0 and vehicle treated APP/E3/Abca1^+/-^ and APP/E4/Abca1^+/-^ mice. N = 4 mice per group. *, p < 0.05, ***, p<0.001.

In validation qPCR assays we confirmed up-regulation of *Abca1*, *Abcg1*, *Scd1* and *Scd2*, *Srebf1* and *Lpcat3* using total RNA isolated from brains of male and female mice of both genotypes (Fig 3C and 3E). To confirm the effect of activated LXR/RXR on transcription is translated into increased protein level, we performed western blotting on ABCA1 and APOE. As visible from Fig 4A, T0 treatment increased ABCA1 protein level in both genotypes. Fig 4B shows that pharmacological LXR/RXR activation by T0 did not change total APOE protein level which is in agreement with gene expression data. Similarly, we did not observe any significant effect of activated LXR/RXR on APOJ/CLU protein level (Fig 4B). Lastly, we examined APOE lipidation using native PAGE. As shown in Fig 4C, T0 treatment increased APOE lipidation in APP/E3/Abca1^+/-^ and APP/E4/Abca1^+/-^ mice of both genders. Interestingly, APOE lipidation in APP/E4/Abca1^+/-^ was significantly lower than in APP/E3/Abca1^+/-^ mice. We conclude that in both isoforms, LXR treatment significantly increased protein level of ABCA1 that in turn affects cholesterol efflux and APOE lipidation.

### APOE isoform-specific effect on gene expression

**Fig 5:**
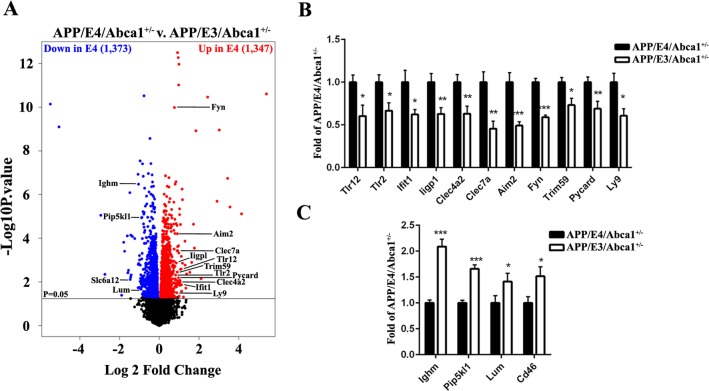
APOE isoform-specific effect on gene expression. **A,** Comparison between APP/E4/Abca1^+/-^ (vehicle plus T0) and APP/E3/Abca1^+/-^ mice (vehicle plus T0). The volcano plot shows differential gene expression between APP/E4/Abca1^+/-^ and APP/E3/Abca1^+/-^ mice. Data were analyzed using EdgeR and the volcano plots are built using p < 0.05 cut-off. Up- and Down-regulated genes are represented in red and blue respectively. On **B** and C are shown genes that are up- or down-regulated in APP/E4/Abca1^+/-^ mice B, shown is RNA-seq result for genes that are significantly upregulated in APP/E4/Abca1^+/-^ vs APP/E3/Abca1^+/-^ mice. **C,** shown is RNA-seq result for genes that are significantly down-regulated in APP/E4/Abca1^+/-^ vs APP/E3/Abca1^+/-^ mice. *, p < 0.05, **, p < 0.01, ***, p < 0.001.

Since PCA (Fig 3A) showed high isoform-dependent variability in APP/E3/Abca1^+/-^ and APP/E4/Abca1^+/-^ datasets, we tested the effect of *APOE* genotype by comparing all APP/E4/Abca1^+/-^ mice (vehicle and T0 treated) to all APP/E3/Abca1^+/-^ mice (vehicle and T0 treated). Using p value at p < 0.05 as a cut-off we identified 1,373 UP- and 1,347 Down-regulated genes in APP/E4/Abca1^+/-^ mice when compared to APP/E3/Abca1^+/-^ (Fig 5A).

To examine biological categories affected by APOE isoform we performed gene ontology analysis using DAVID. We used significantly up- or down-regulated genes at p < 0.05 and Log2 fold change higher than 0.5. As shown on S3 Table, significantly up-regulated in APP/E4/Abca1^+/-^ were categories such as innate immune response, response to interferon β, response to cytokines and cell proliferation. Genes that are members of this category such as toll-like receptors (*Tlr12* and *Tlr2*), C-type lectin domain genes (*Clec7a/Dectin1* and *Clec4a2*), interferon inducible genes (*Ifit1* and *Iigp1*), FYN proto-oncogene, Src family tyrosine kinase (*Fyn)* [40] and others are shown on Fig 5B. Down-regulated were categories such as visual perception (examples are genes such as *Lum*, lumican) and phagocytosis (*Ighm*, immunoglobulin heavy constant mu, *Cd46/Mcp* [41], *Pip5kl1* [42]) are shown on Fig 5C.

### T0 differentially affects gene expression in APP/E4/Abca1^+/-^ and APP/E3/Abca1^+/-^ mice

**Fig 6:**
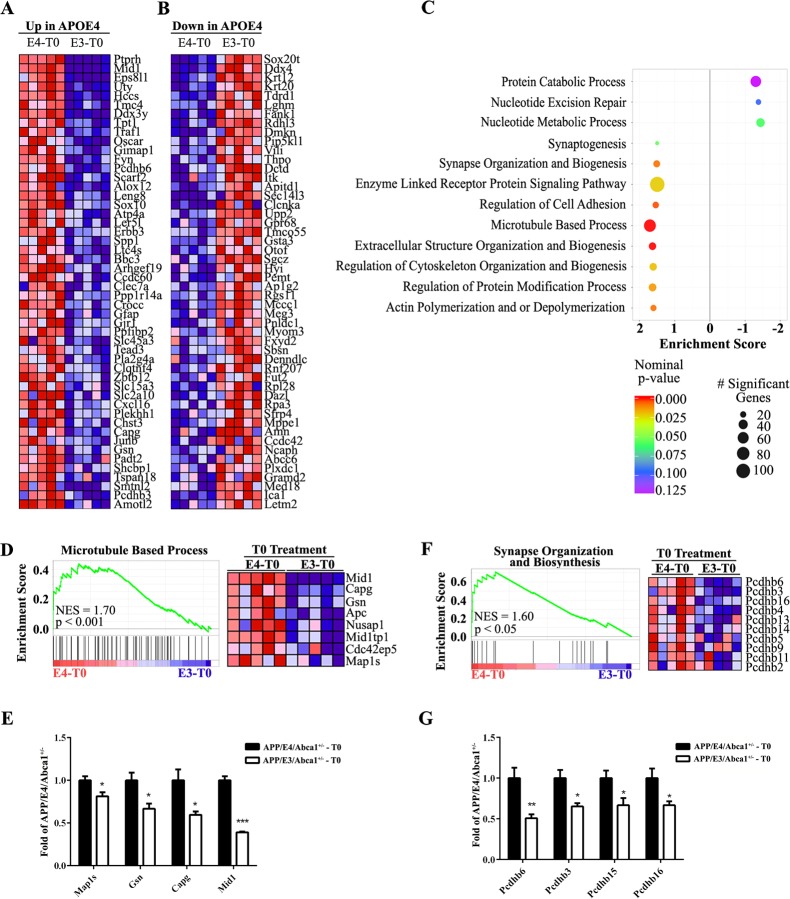
APOE isoform-specific effect on gene expression in APP/E3/Abca1^+/-^ and APP/E4/Abca1^+/-^ mice. Comparison between T0 treated APP/E4/Abca1^+/-^ and APP/E3/Abca1^+/-^ mice. Heat-maps provided by GSEA analysis were used to identify and rank the top 50 up-regulated genes (**A**) and top 50 down regulated genes (**B**) in APP/E4/Abca1^+/-^ mice. **C,** Bubble plot shows top ranked “biological process” (BP) differentially affected by T0 treatment in APP/E4/Abca1^+/-^ vs APP/E3/Abca1^+/-^ mice. The gene lists were derived from edgeR output tables and included expression data for all transcripts. Color indicates nominalized p-value. Significant BP are represented in red to purple shades (p<0.05 and FDR≤0.25). Size of bubble indicates the number of significant genes in each represented BP. GSEA enrichment score curves and corresponding heat-maps show BP significantly enriched in T0 treated APP/E4/Abca1^+/-^ mice, **D** and **E,** “Microtubule Based Process”. **D,** GSEA analysis provided a heat-map (right) and enrichment score (left) for this category. **E,** RNA-seq results of significantly changed mRNA expression levels of representative genes from category “Microtubule Based Process”. **F-G,** “Synapse Organization and Biosynthesis”. **F,** GSEA analysis provided a heat-map (right) and enrichment score (left). **G,** RNA-seq results of significantly changed mRNA expression levels of representative genes from category “Synapse Organization and Biosynthesis”. *, p < 0.05, **, p < 0.01, ***, p < 0.001.

Next, to examine if LXR treatment differentially affects biological processes in isoform-dependent manner, we applied Gene Set Enrichment Analysis (GSEA) and compared T0 treated APP/E4/Abca1^+/-^ and APP/E3/Abca1^+/-^ mice. We included expression data for all transcripts without setting a cut-off to avoid a bias towards the effect of highly affected genes [39]. Using GSEA, we ranked the top 50 up- (Fig 6A) and down-regulated (Fig 6B) genes of the gene-ontology category “biological process”. The bubble plot shown on Fig 6C represents biological processes affected by T0 treatment in APP/E3/Abca1^+/-^ (right) and APP/E4/Abca1^+/-^ mice (left) and is based on the number of genes in each category and the nominal p-value (see also S4 Table). To further illustrate significantly enriched biological process terms, we show enrichment plots with corresponding heat maps for “Microtubule Based Process” and “Synapse Organization and Biosynthesis”. While the morphological and functional validation of the affected biological processes in APP/E4/Abca1^+/-^ is beyond the scope of this study, the results are suggesting APOE isoform specific response to LXR/RXR activation and enrichment in sets of genes that help to better understand positive effects of treatment on cognitive performance.

## Discussion

In this study we analyzed the effect of LXR agonist T0 on the phenotype of *Abca1* haplo-deficient APP/E3 and APP/E4 mice. The results demonstrate that T0 treatment significantly ameliorates cognitive deficits seen in APP/E4/Abca1^+/-^ mice, as examined by Novel Object Recognition and Contextual Fear Conditioning paradigms. T0 treatment also reduced soluble Aβ oligomers without affecting amyloid plaques, confirming our recent study [34]. Importantly, RNA-seq results and the analysis of changes in brain transcriptome demonstrated that commonly up-regulated genes in response to T0 induced LXR/RXR activation affect lipoprotein metabolism and APOE lipidation.

Prior studies have demonstrated that treatment with LXR agonists ameliorates memory deficits in APP mice [18, 23, 34, 43–46]. We postulate that the increased lipidation of APOE can affect the phenotype through an increased clearance of Aβ oligomers and an increased supply of cholesterol and phospholipids to neurons–“trophic effect”. As seen on the Native-PAGE, T0 increases the level of lipidated APOE in cortical homogenates of both APP/E3/Abca1^+/-^ and APP/E4/Abca1^+/-^ mice (Fig 4). Based on our transcriptomics and expression validation data we posit that the increased APOE lipidation is a result of the upregulated expression of *Abca1*, *Abcg1*, *Scd1*, *Scd2* and *Lpcat3* genes, essential for cholesterol efflux. These genes were identified as commonly up-regulated in brain of mice expressing either APOE isoform.

We hypothesize that clearance of Aβ oligomers is an important consequence of the increased level of fully lipidated APOE. APOE-containing lipoproteins could affect Aβ metabolism by decreasing Aβ aggregation and preventing its conversion to oligomers. This in turn maintains Aβ in soluble state and facilitates its clearance by glia or via BBB. In a recent study we showed that lack of *Abca1* significantly decreased Aβ clearance out of the brain and increased its level in ISF [36]. Previous data from our group demonstrated also that treatment of primary WT astrocytes with T0 increased the lipidation of APOE [45]. In the same study, we also showed that fully lipidated APOE-lipoproteins facilitated Aβ42 degradation by microglia in contrast to APOE-lipoproteins isolated from Abca1^-/-^ astrocytes. Furthermore, a study by Jiang et al. demonstrated that *in vitro*, APOE promotes Aβ degradation by microglia and the process is dependent on the APOE isoform and its lipidation [17]. Whereas in this study we did not specifically address the mechanism by which Aβ clearance is affected, our data clearly show that the levels of Aβ oligomers in T0-treated APP/E4/Abca1^+/-^ mice are decreased. Published data from our group also demonstrated that LXR/RXR agonists decrease the level of soluble Aβ40 and Aβ42 in ISF [26, 34, 45], and the treatment of APP/E3 and APP/E4 mice with RXR ligand bexarotene decreases the level of soluble Aβ oligomers in brain parenchyma. Altogether, these data suggest that the effect of T0 treatment on clearance of Aβ soluble species could be a result of concerted action of activated LXRs/RXRs heterodimers. In contrast, here and in previous studies we did not observe an effect on insoluble amyloid deposits after treatment with LXR or RXR ligands [26, 34].

A second consequence of the increased APOE lipidation is that properly lipidated APOE delivers cholesterol and phospholipids to neurons more efficiently. The lipid molecules are needed for repair of axonal/neuronal damage resulting from amyloid deposition and thus for improved synaptic transmission. As extensively discussed in our previous study [36] mice lacking *Apoe* have impairments in cognition and dendritic arborization. Synaptic dysfunction in AD pathogenesis is recognized as an important mechanism and the role of APOE in affecting synaptic plasticity in an isoform-dependent manner has been repeatedly confirmed (reviewed in [6, 47, 48]). As recently shown by our group, treatment of APOE3 or APOE4 mice with RXR ligand bexarotene improved neuronal complexity and increased neuritogenesis in *APOE4* and *APOE3* mice [31, 32].

Our results also show that there is a significant APOE isoform-specific effect on expression of genes with a potential to affect amyloid phenotype and behavior. For example, mRNA expression of genes related to innate immune response such as toll-like receptors, *Fyn* and interferon related genes were higher in APP/E4/Abca1^+/-^ than in APP/E3/Abca1^+/-^ mice (Fig 5B). Some of these genes such as *Tlr2* [49] and *Fyn* [50] were shown to affect cognitive performance, a result that correlates with the APP/E4/Abca1^+/-^ mice performing worse in cognitive tests (Fig 1). Interestingly, phagocytosis was a category significantly down-regulated in APP/E4/Abca1^+/-^ mice.

When we compared only agonist treated APP/E4/Abca1^+/-^ and APP/E3/Abca1^+/-^ mice, we identified “Microtubule Based Process” and “Synapse Organization and Biosynthesis” as GO categories uniquely enriched in APP/E4/Abca1^+/-^ mice. Members of the beta-protocadherin (*Pcdh-β*) family were up-regulated in T0 treated APP/E4/Abca1^+/-^ mice. *Pcdh* genes, a subfamily of cadherin adhesion molecules, are expressed in the brain (reviewed in [51]) and have been demonstrated essential in establishing functional synapses [52]. *Pcdh* gene family expression has been identified in various neuronal populations and the protein localizes predominantly in synapses. An isoform of *Pcdhg-β*, *Pcdhβ-16* is expressed in the hippocampus and cortical layers [52] and we found isoforms of *Pcdhβ-16* up-regulated following T0 treatment. Although currently the research focuses primarily on PCDH-α and PCDH-γ and their ability to mediate cell adhesion through combinatorial expression on the surface of neurons [53, 54], it is reasonable to assume that PCDH-β could be involved in those processes, as well. PCDH-β can localize to synapses, suggesting the protein might have the potential to contribute to the formation of synaptic plasticity in the mammalian CNS. No research, however, has been conducted so far, to reveal if their function is interconnected to APOE secretion and deposition of Aβ, or cholesterol transport and its internalization at the synaptic level, or the way they influence the cholesterol/phospholipid composition of the cell membrane, necessary and required for normal neuronal function.

In conclusion, the present findings show that LXR agonist treatment of *Abca1* haplo-deficient APP/E4 mice, ameliorates APOE4 driven brain pathology and cognitive deficits. The results are attributed to the ability of T0, through LXR/RXR activation, to reverse lipid deficiency of APOE4 particles in brain. The results of our study also suggest that an increased ABCA1 and ABCG1 expression through LXR/RXR activation, resulting in improved APOE lipidation, may be a useful target for future prophylactic as well as therapeutic approaches in *APOE4* carriers.

## Supporting information

S1 Table Gene ontology categories (GO) UP-regulated in T0 treated APP/E3/Abca1^+/-^ and APP/E4/Abca1^+/-^mice. (PDF)

S2 Table Gene ontology categories (GO) Down-regulated in T0 treated APP/E3/Abca1^+/-^ and APP/E4/Abca1^+/-^mice. (PDF)

S3 Table (A, B). Gene ontology categories (GO) UP-regulated in APP/E4/Abca1^+/-^mice.
(PDF)

S4 Table Top 20 up- and down-regulated GSEA Biological Process in APP/E4/Abca1^+/-^ vs APP/E3/Abca1^+/-^ T0 treated mice. (PDF)
