# Supplementary material for: Liver X receptor agonist treatment significantly affects phenotype and transcriptome of APOE3 and APOE4 Abca1 haplo-deficient mice
Source: PLoS One. 2017 Feb 27;12(2):e0172161. doi: 10.1371/journal.pone.0172161 (PMC5328633; doi:10.1371/journal.pone.0172161)
Supplement: S1 Table — (PDF) [file pone.0172161.s001.pdf]

S1 Table. Gene ontology categories (GO) UP-regulated in T0 treated APP/E3/Abca1$^{+/-}$ and APP/E4/Abca1$^{+/-}$ mice.

**APP/E3/Abca1$^{+/-}$: T0 vs Vehicle**					
Term	Count	%	PValue	FE	Benjamini
**GO:0006629~lipid metabolic process**[a]	12	8.51	1.88E-04	4.01	0.11
**GO:0006633~fatty acid biosynthetic process**	5	3.55	0.001	10.84	0.22
**GO:0055091~phospholipid homeostasis**	3	2.13	0.001	49.88	0.22
**GO:0006281~DNA repair** [b]	8	5.67	0.004	3.94	0.32
**GO:0042632~cholesterol homeostasis**	4	2.84	0.007	9.98	0.45
GO:0006310~DNA recombination	4	2.84	0.015	7.58	0.64
**GO:0006974~cellular response to DNA damage stimulus**	8	5.67	0.018	2.94	0.67
**GO:0016568~chromatin modification**	6	4.26	0.032	3.36	0.74
**APP/E4/Abca1$^{+/-}$: T0 vs Vehicle**					
Term	Count	%	PValue	FE	Benjamini
GO:0006355~regulation of transcription, DNA-templated	69	14.90	2.50E-05	1.66	0.04
**GO:0006974~cellular response to DNA damage stimulus**	20	4.32	7.88E-04	2.39	0.43
GO:0006351~transcription, DNA-templated	57	12.31	0.001	1.52	0.46
**GO:0006281~DNA repair**	14	3.02	0.010	2.24	0.92
**GO:0006633~fatty acid biosynthetic process**	6	1.30	0.013	4.22	0.94
GO:0006810~transport	50	10.80	0.020	1.36	0.95
GO:0000723~telomere maintenance	4	0.86	0.034	5.55	0.98
**GO:0016568~chromatin modification**	11	2.38	0.049	2.00	0.99

[a], In Red are shown pathways related to lipid and cholesterol metabolism;

[b], In bold are marked GO terms overlapping in both APOE isoforms
