# Supplementary material for: Liver X receptor agonist treatment significantly affects phenotype and transcriptome of APOE3 and APOE4 Abca1 haplo-deficient mice
Source: PLoS One. 2017 Feb 27;12(2):e0172161. doi: 10.1371/journal.pone.0172161 (PMC5328633; doi:10.1371/journal.pone.0172161)
Supplement: S2 Table — (PDF) [file pone.0172161.s002.pdf]

S2 Table. Gene ontology categories (GO) Down-regulated in T0 treated APP/E3/Abca1$^{+/-}$ and APP/E4/Abca1$^{+/-}$ mice.

APP/E3/Abca1$^{+/-}$: T0 vs Vehicle					
Term	Count	%	PValue	FE	Benjamini
GO:0007155~cell adhesion [a]	18	6.19	3.48E-04	2.72	0.40
GO:0045087~innate immune response	15	5.15	4.11E-04	3.06	0.26
GO:0043410~positive regulation of MAPK cascade	8	2.75	6.72E-04	5.44	0.28
GO:0042127~regulation of cell proliferation	11	3.78	9.34E-04	3.63	0.24
**GO:0043065~positive regulation of apoptotic process[b]**	13	4.47	0.001	3.00	0.29
GO:0005977~glycogen metabolic process	5	1.72	0.001	10.10	0.26
GO:0030335~positive regulation of cell migration	10	3.44	0.001	3.70	0.25
GO:0008284~positive regulation of cell proliferation	15	5.15	0.004	2.35	0.45
GO:0035458~cellular response to interferon-beta	4	1.37	0.008	9.42	0.52
GO:0055114~oxidation-reduction process	18	6.19	0.010	1.95	0.53
**APP/E4/Abca1$^{+/-}$: T0 vs Vehicle**					
Term	Count	%	PValue	FE	Benjamini
GO:0009968~negative regulation of signal transduction	7	2.16	1.45E-04	8.67	0.18
GO:0030512~negative regulation of transforming growth factor beta receptor signaling pathway	6	1.85	0.001	7.43	0.57
GO:0030154~cell differentiation	23	7.10	0.002	2.01	0.59
GO:0032436~positive regulation of proteasomal ubiquitin-dependent protein catabolic process	6	1.85	0.003	6.06	0.57
GO:0006469~negative regulation of protein kinase activity	6	1.85	0.014	4.19	0.92
GO:0090090~negative regulation of canonical Wnt signaling pathway	6	1.85	0.015	4.10	0.91
**GO:1901214~regulation of neuron death [a]**	3	0.93	0.020	13.13	0.91
GO:0060326~cell chemotaxis	4	1.23	0.034	5.59	0.94
GO:0007268~synaptic transmission	6	1.85	0.036	3.28	0.94
GO:0046777~protein autophosphorylation	7	2.16	0.049	2.64	0.97

[a], In bold are marked GO terms overlapping in both APOE isoforms.
