# Supplementary material for: Liver X receptor agonist treatment significantly affects phenotype and transcriptome of APOE3 and APOE4 Abca1 haplo-deficient mice
Source: PLoS One. 2017 Feb 27;12(2):e0172161. doi: 10.1371/journal.pone.0172161 (PMC5328633; doi:10.1371/journal.pone.0172161)
Supplement: S3 Table — (A, B). Gene ontology categories (GO) UP-regulated in APP/E4/Abca1+/-mice. (PDF) [file pone.0172161.s003.pdf]

S3A Table. Gene ontology categories (GO) UP-regulated in APP/E4/Abca1$^{+/-}$mice.

Term	Count	%	PValue	FE	Benjamini
GO:0035458~cellular response to interferon-beta	5	2.87	2.36E-04	16.33	0.18
GO:0042127~regulation of cell proliferation	9	5.17	2.93E-04	5.31	0.12
GO:2001056~positive regulation of cysteine-type endopeptidase activity	3	1.72	5.37E-04	80.36	0.14
GO:0045087~innate immune response	11	6.32	8.02E-04	3.68	0.16
GO:0010466~negative regulation of peptidase activity	6	3.45	0.002	6.87	0.26
GO:0002376~immune system process	10	5.75	0.002	3.50	0.27
GO:0034097~response to cytokine	5	2.87	0.003	8.27	0.31

S3B Table. Gene ontology categories (GO) DOWN-regulated in APP/E4/Abca1$^{+/-}$mice.

Term	Count	%	PValue	FE	Benjamini
GO:0007601~visual perception	6	3.53	7.84E-04	8.24	0.32
GO:0006958~complement activation, classical pathway	4	2.35	0.003	13.28	0.56
GO:0006910~phagocytosis, recognition	3	1.76	0.018	14.42	0.83
GO:0006911~phagocytosis, engulfment	3	1.76	0.02	12.18	0.87
GO:0031047~gene silencing by RNA	3	1.76	0.03	10.34	0.91
GO:0008152~metabolic process	7	4.12	0.04	2.76	0.89
