# Supplementary material for: Liver X receptor agonist treatment significantly affects phenotype and transcriptome of APOE3 and APOE4 Abca1 haplo-deficient mice
Source: PLoS One. 2017 Feb 27;12(2):e0172161. doi: 10.1371/journal.pone.0172161 (PMC5328633; doi:10.1371/journal.pone.0172161)
Supplement: S4 Table — (PDF) [file pone.0172161.s004.pdf]

S4 Table. Top 20 up- and down-regulated GSEA Biological Process in APP/E4/Abca1$^{+/-}$ vs APP/E3/Abca1$^{+/-}$ T0 treated mice.

UP regulated in APP/E4/Abca1$^{+/-}$ vs APP/E3/Abca1$^{+/-}$				
NAME	SIZE	NES	NOM p-val	FDR q-val
**MICROTUBULE_CYTOSKELETON_ORGANIZATION_AND_BIOGENESIS [a]**	**31**	**-1.87**	**0.00**	**0.16**
**NEGATIVE_REGULATION_OF_PROTEIN_METABOLIC_PROCESS**	**31**	**-1.68**	**0.01**	**0.24**
ANTI_APOPTOSIS	89	-1.64	0.00	0.31
REGULATION_OF_PROTEIN_MODIFICATION_PROCESS	28	-1.63	0.01	0.26
**SYNAPSE_ORGANIZATION_AND_BIOGENESIS [b]**	**20**	**-1.60**	**0.01**	**0.25**
ONE_CARBON_COMPOUND_METABOLIC_PROCESS	25	-1.55	0.04	0.37
REGULATION_OF_ORGANELLE_ORGANIZATION_AND_BIOGENESIS	35	-1.54	0.02	0.38
ACTIN_POLYMERIZATION_AND_OR_DEPOLYMERIZATION	21	-1.54	0.00	0.36
REGULATION_OF_CELL_ADHESION	23	-1.51	0.01	0.40
PEPTIDYL_TYROSINE_PHOSPHORYLATION	16	-1.49	0.04	0.39
CELL_CYCLE_ARREST_GO_0007050	46	-1.49	0.05	0.38
NEGATIVE_REGULATION_OF_PROGRAMMED_CELL_DEATH	112	-1.49	0.03	0.36
**Down regulated in APP/E4/Abca1$^{+/-}$ vs APP/E3/Abca1$^{+/-}$**				
NAME	SIZE	NES	NOM p-val	FDR q-val
NUCLEOTIDE_METABOLIC_PROCESS	34	1.44	0.06	1
NUCLEOTIDE_EXCISION_REPAIR	18	1.38	0.10	1
PROTEIN_CATABOLIC_PROCESS	55	1.31	0.13	1
TRANSCRIPTION_INITIATION	24	1.27	0.18	1
PHOSPHOLIPID_METABOLIC_PROCESS	55	1.24	0.11	1
STRESS_ACTIVATED_PROTEIN_KINASE_SIGNALING_PATHWAY	41	1.19	0.18	1
REGULATION_OF_DNA_METABOLIC_PROCESS	36	1.16	0.28	1
PROTEIN_MODIFICATION_BY_SMALL_PROTEIN_CONJUGATION	39	1.15	0.26	1
PROTEIN_DNA_COMPLEX_ASSEMBLY	36	1.15	0.27	1
NEGATIVE_REGULATION_OF_MAP_KINASE_ACTIVITY	16	1.12	0.27	1
JNK_CASCADE	39	1.11	0.32	1
MEMBRANE_LIPID_METABOLIC_PROCESS	79	1.09	0.31	1

[a], With bold are marked statistically significant Biological Process (BP) (FDR≤0.25)
